# Supplementary material for: Tumor invasion in draining lymph nodes is associated with Treg accumulation in breast cancer patients
Source: Nat Commun. 2020 Jun 29;11:3272. doi: 10.1038/s41467-020-17046-2 (PMC7324591; doi:10.1038/s41467-020-17046-2)
Supplement: Supplementary file 1 — Supplementary Information [file 41467_2020_17046_MOESM1_ESM.pdf]

SUPPLEMENTARY INFORMATION

Tumor invasion in draining lymph nodes is associated with Treg accumulation in breast cancer patients

Nicolas Gonzalo Núñez, Jimena Tosello et al.

Corresponding author:
Eliane Piaggio. E-mail: eliane.piaggio@curie.fr

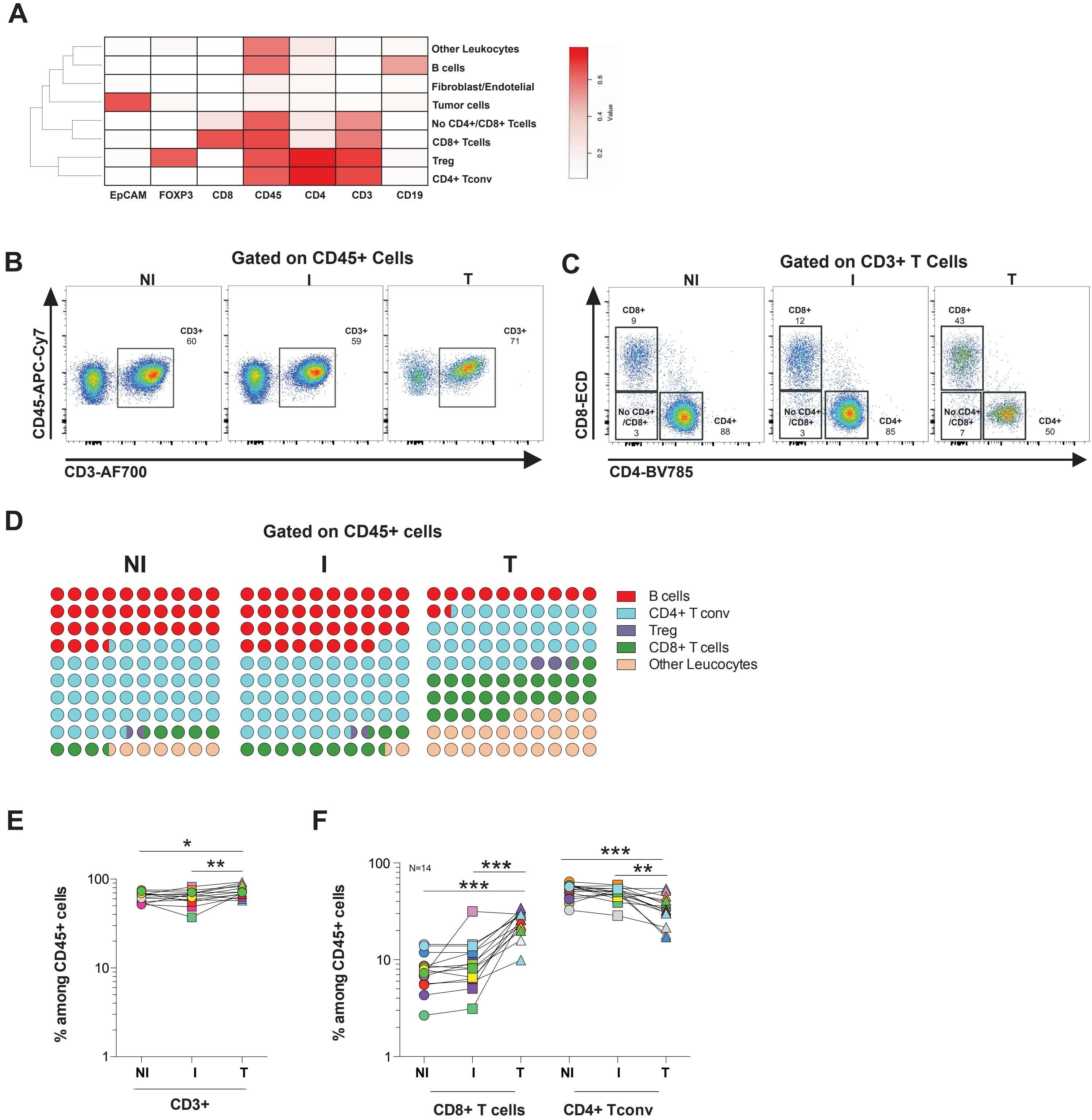

Supplementary Figure 1. Overall distribution of immune cell populations in NI and I TDLNs, and tumor. Cell suspensions of TDLNs and tumors were stained for CD45, CD3, CD4 and CD8. A) Heat map showing the median marker expression value for each defined population from Figure 1E. Red and white indicate higher and lower expression, respectively. B) Representative flow cytometric analysis of CD3+ T cells among CD45+ cells. C) Representative flow cytometric analysis of CD4+ and CD8+ T cells among CD3+ T cells. D) Frequencies of B cell and T cell subpopulations among CD45+ cells in TDLNs and tumor. E) Frequency of T cells among CD45+ cells in TDLNs and tumor (NI TDLN vs T p=0.017; I TDLN vs T p=0.0037). Wilcoxon matched-pairs signed rank test. (N=13). F) Frequency of CD8+ and CD4+ Tconvs among CD45+ cells in TDLNs and tumor (CD8+ T cells: NI TDLN vs T p=0.0001; I TDLN vs T p=0.0002; CD4+ Tconv: NI TDLN vs T p=0.0006; I TDLN vs T p=0.0012). Wilcoxon matched-pairs signed rank test, *, p < 0.05, **, p < 0.01, ***, p < 0.001. (N=14).

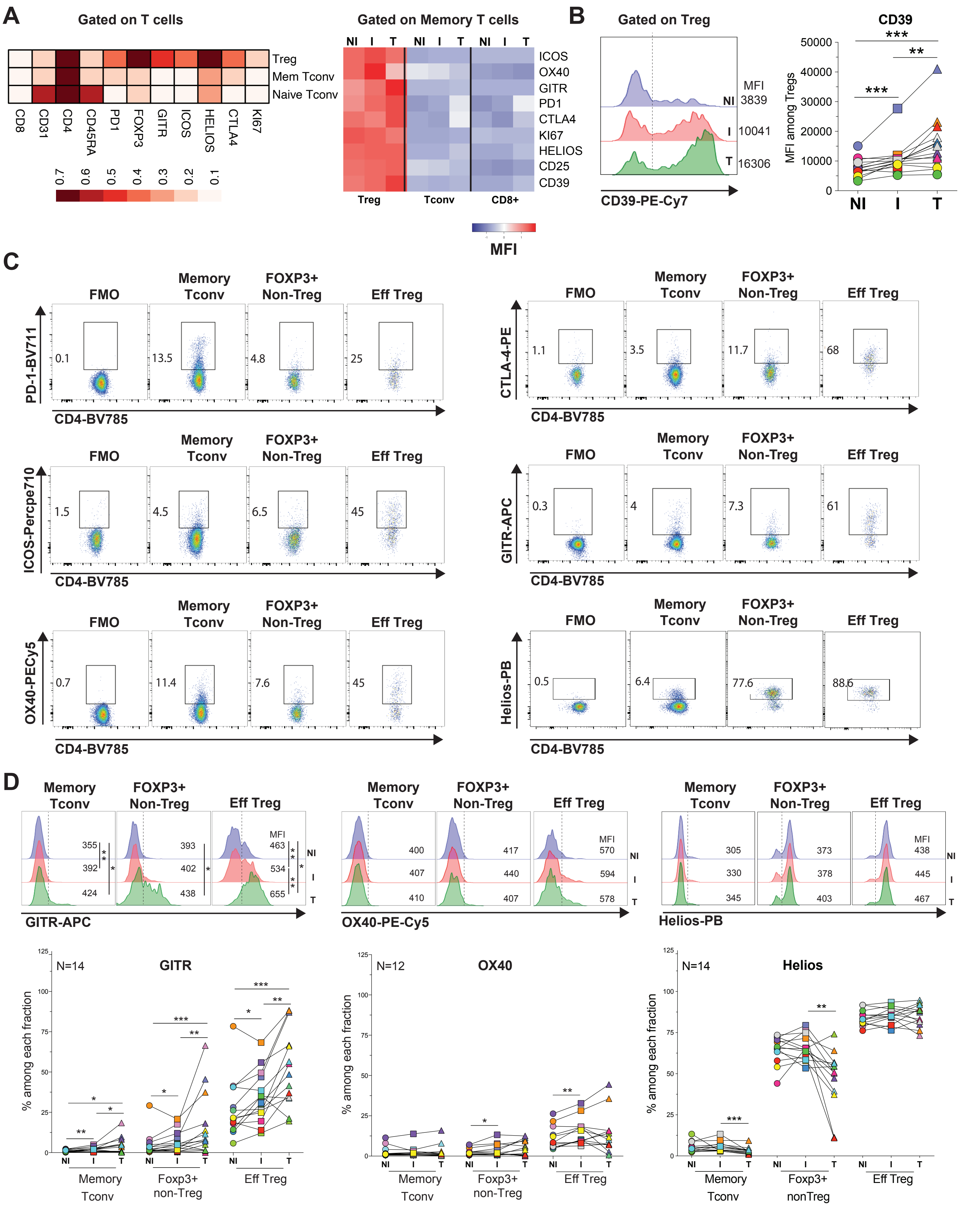

Supplementary Figure 2. Immune checkpoint expression profile of CD4+T cells from TDLNs and tumor. Cell suspensions of TDLNs and tumors were stained for CD3, CD4, CD45RA, FOXP3, ICOS, GITR, OX40, PD-1, CTLA-4, CD31, CD25, CD39, HELIOS and KI67. A) Heat maps showing the median marker expression value for each defined population (left panel) or each tissue (right panel) from Figure 2A. Red and blue indicate higher and lower expression, respectively. B) Representative flow cytometric analysis and MFIs for CD39 among Eff Tregs in TDLNs and tumor. NI vs I TDLNs p=0.0009; NI TDLN vs T p=0.0001; I TDLN vs T p=0.0012. Wilcoxon matched-pairs signed rank test .(N=14). C) Representative flow cytometric analysis and MFIs for PD-1, CTLA-4, ICOS, GITR, OX40 and Helios among the indicated memory CD4+ T cell subpopulations. D) Representative histograms and frequencies of GITR (Memory Tconv: NI vs I TDLNs p=0.0031; NI TDLN vs T p=0.0101; I TDLN vs T p=0.0494; Foxp3+non-Treg: NI vs I TDLNs p=0.0419; NI TDLN vs T p=0.0009; I TDLN vs T p=0.0052; Eff Treg: NI vs I TDLNs p=0.0245; NI TDLN vs T p=0.0001; I TDLN vs T p=0.0017), OX40 (Foxp3+non-Treg: NI vs I TDLNs p=0.0425; Eff Treg: NI vs I TDLNs p=0.0024) and Helios (Memory Tconv: I TDLN vs T p=0.0005; Foxp3+non-Treg: I TDLN vs T p=0.0024) among the indicated memory CD4+ T cell subpopulations in TDLNs and tumor.(N=14). Wilcoxon matched-pairs signed rank test, *, $p < 0.05$, **, $p < 0.01$, ***, $p < 0.001$.

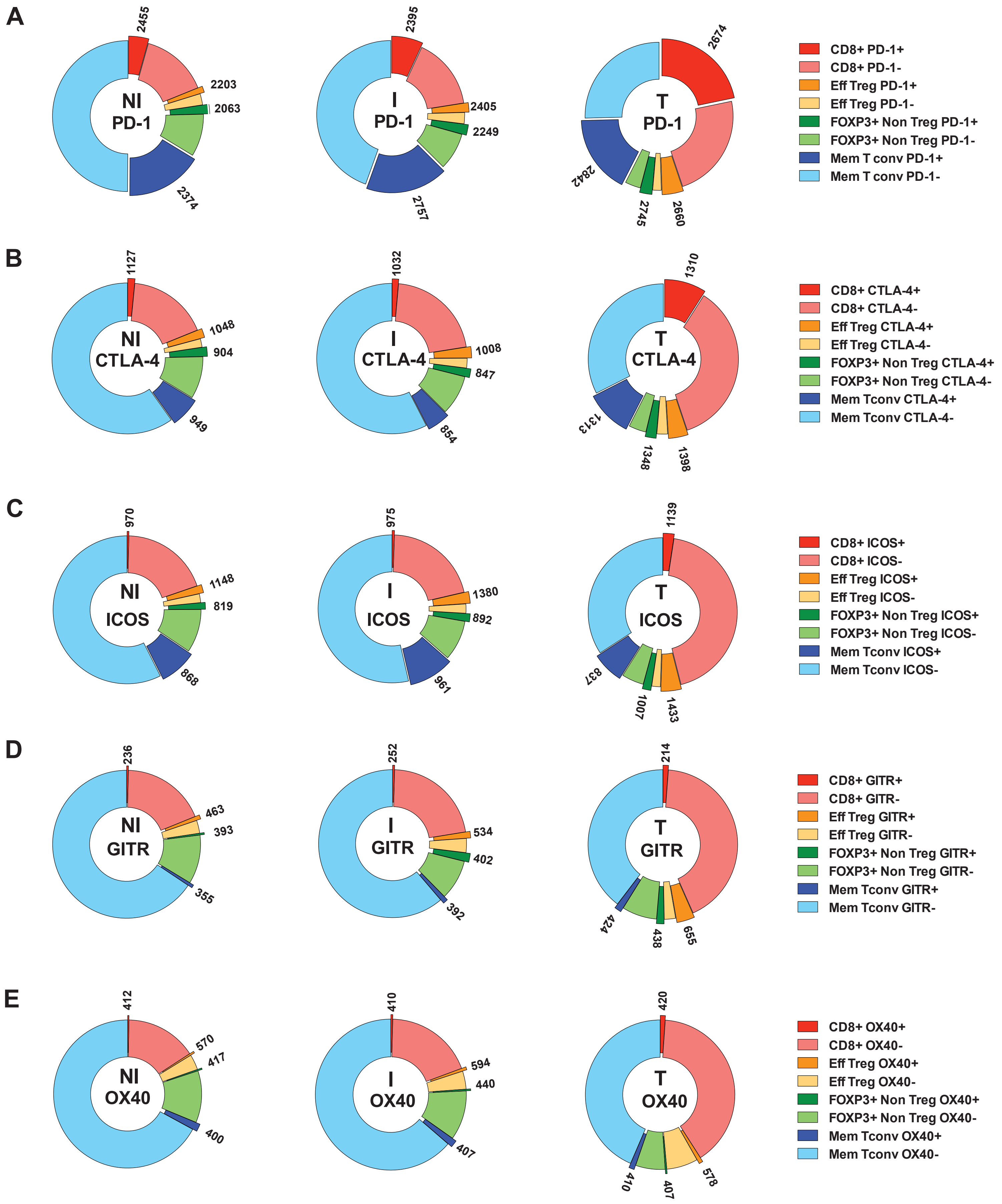

Supplementary Figure 3. Immune checkpoint expression profile of T cells from TDLNs, and tumor. Pie charts illustrating the relative size of CD8+ T cells, effector Tregs, FOXP3+ Non Treg (activated Tconvs) and, memory Tconvs cell populations expressing PD-1(A), CTLA-4(B), ICOS(C), GITR(D), and OX40(E). Exploded and darker slices represent the positive and target populations. The data are quoted as the mean MFI for each T cell subpopulation (N=12).

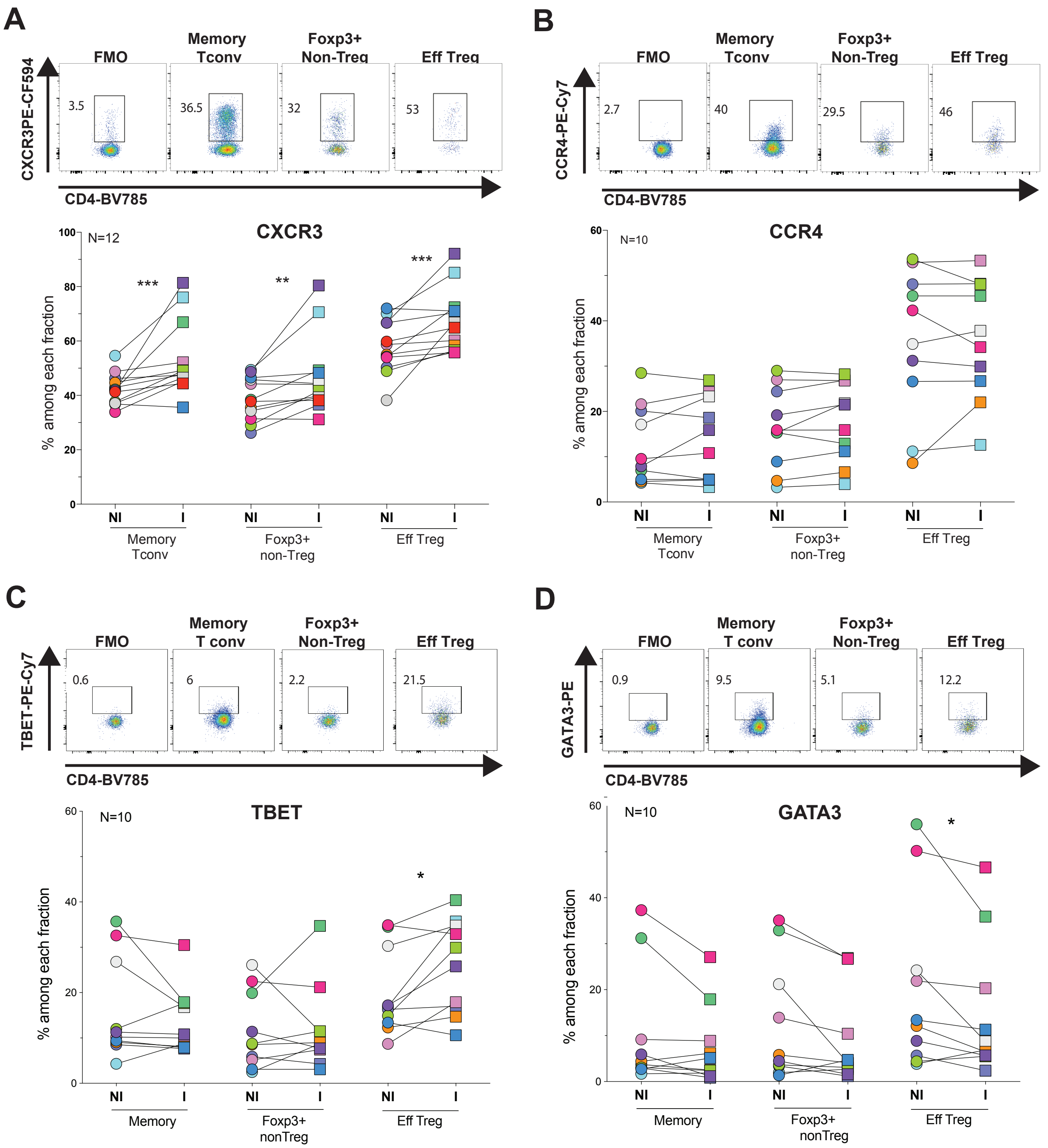

Supplementary Figure 4. CD4+ T cell chemokine receptors and transcription factors in TDLNs. Cell suspensions of TDLNs were stained for CD3, CD4, CD45RA, FOXP3, CXCR3, CCR4, Tbet and Gata3. Representative flow cytometric analysis (upper panel) and frequency (lower panel) of (A) CXCR3 (Memory Tconv: p=0.0010; Foxp3+non-Treg: p=0.0088; Eff Treg: p=0.0010 in NI vs I TDLNs), (B) CCR4 , (C) Tbet (Eff Treg: p=0.0273 in NI vs I TDLNs) and (D) Gata3 (Eff Treg: p=0.0195 in NI vs I TDLNs)among gated CD4+ T cell subpopulations (N=12) in TDLNs. Wilcoxon matched-pairs signed rank test, *, P < 0.05, **, P < 0.01, ***, P < 0.001

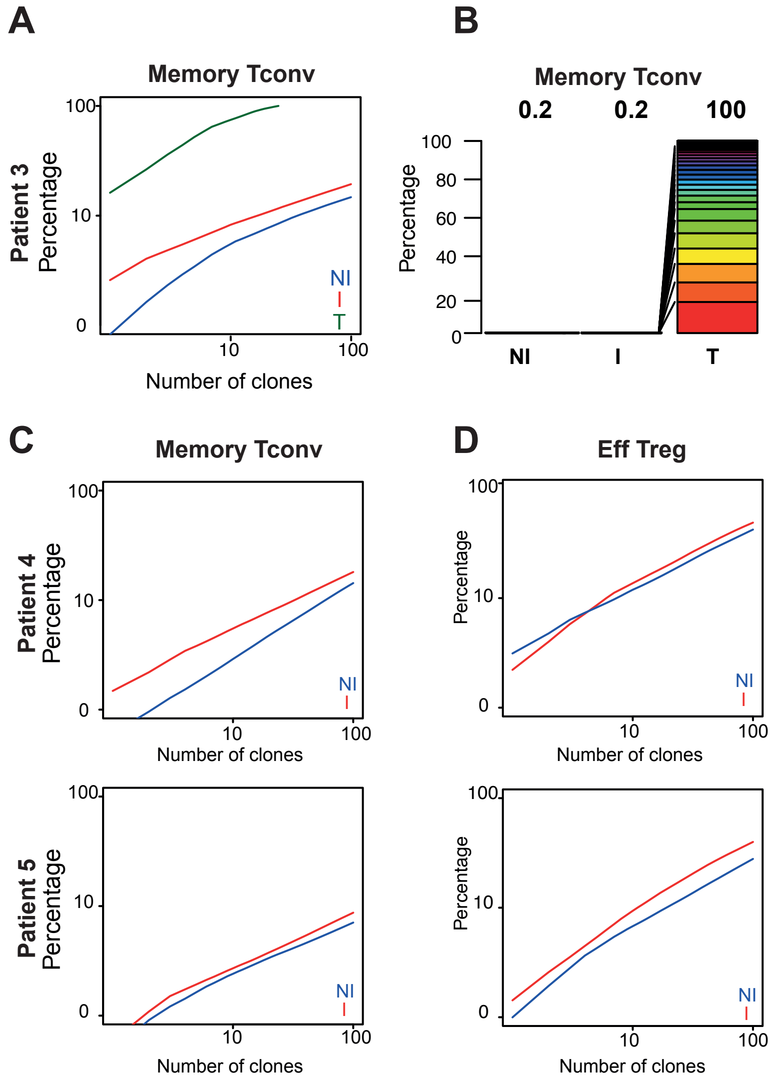

Supplementary Figure 5. TCR repertoire of Tconvs and Tregs from TDLNs, and tumor. Next-generation-sequencing-based high-throughput TCR-β CDR3 analysis of bulk sorted memory T CD4+ Tconvs and Tregs from matched NI and I TDLNs and the primary tumor. A, C-D) Graphs show cumulative frequencies of TCR-β CDR3 clones of memory Tconv or Tregs from NI TDLNs (blue), I TDLNs (red) and the corresponding primary tumor when available (green), for three patients. B) Stacked bar chart depicting the usage frequencies of the top 100 tumor TCR-β CDR3 sequences and their distribution in matched NI and I TDLNs shown for Tconvs, for one patient. Numbers in the graphs indicate percentage of shown TCR-β CDR3 sequences out of total TCR-β CDR3 sequences in the sample.

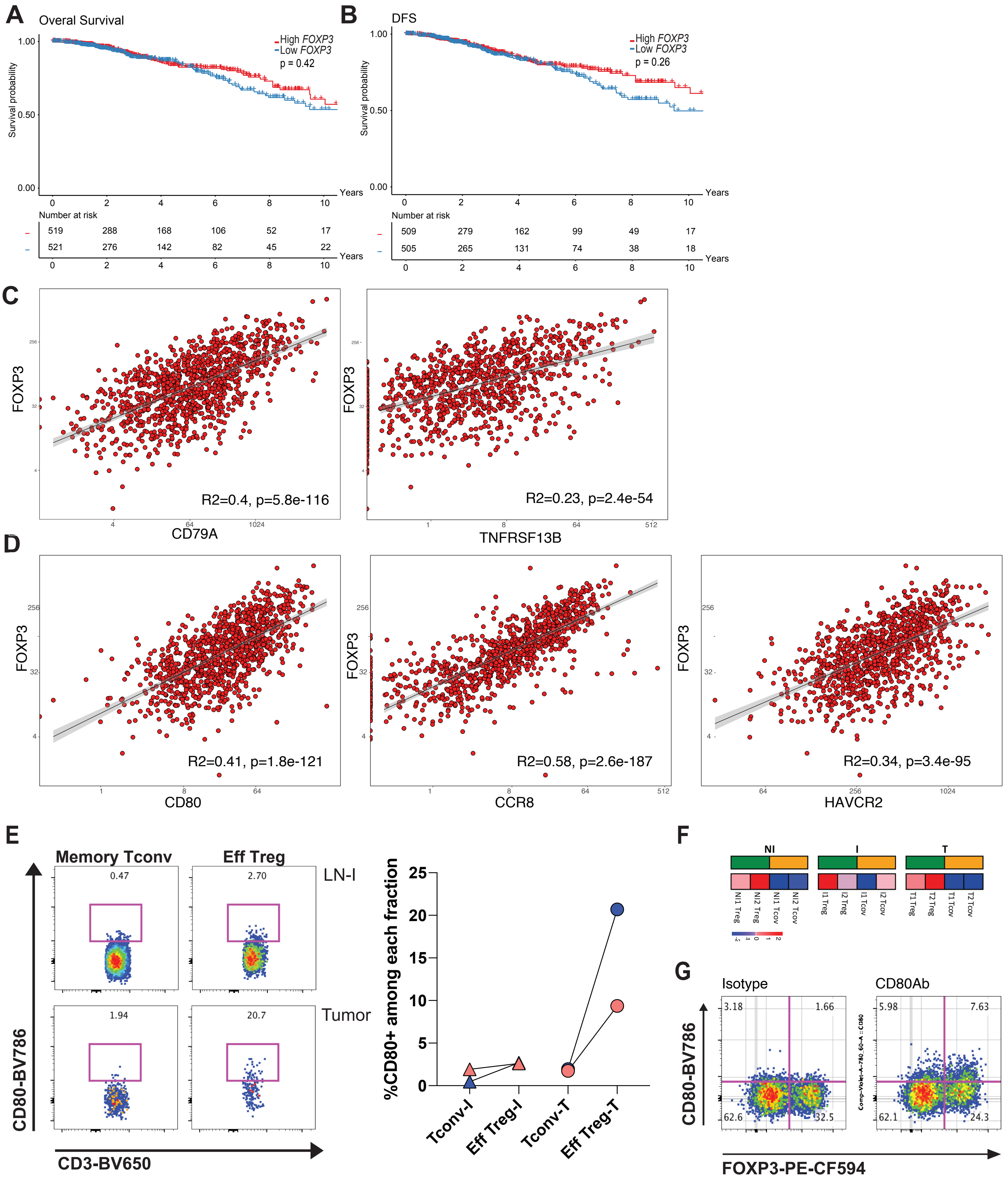

Supplementary Figure 6. Clinical outcome of DEGs normalized to FOXP3 and CD80 expression. OS (A) and DFS (B) of patients with breast cancer stratified by high or low median intensity of the *FOXP3* gene. (C) Scatter plots and linear regression graphs of the correlation between the reads per million (RPM) of *CD79A, TNFRSF13B* genes vs *FOXP3* gene. (D) Scatter plots and linear regression graphs of the correlation between the reads per million (RPM) of *CD80, CCR8* and *HAVCR2* genes vs *FOXP3* gene. E) Representative flow cytometric analysis (left panel) and frequency (right panel) of CD80 expression on Tconv and Treg cells from I TDLNs or tumor (N=2). F) Heatmaps showing CD80 gene expression on Tregs and Tconvs from the 3 tissues. G) Representative dot plot of CD80 expression versus FOXP3 expression on gated CD4+ T cells from tumor.

Supplementary Table 1. Clinical and pathological data of the luminal breast cancer patients

Characteristics	Patients (N=54)	Value
Age (years)		57 ± 14
**Lymph node status**		
Negative (N0)	0	0%
N1 (1–3)	30	56%
N2 (4–9)	14	26%
N3 (>9)	10	19%
**Stage**		
I	0	0%
II	26	48%
III	27	50%
IV	1	2%
**Histology**		
Invasive ductal carcinoma (IDC)	42	78%
Invasive lobular carcinoma (ILC)	8	15%
Others (papillary, medullary, mucinous)	4	7%
**Tumor size (cm)**		
T1 (≤2)	16	30%
T2 (2–5)	28	52%
T3 (>5)	10	19%
**Hormone receptor status**		
Estrogen receptor (ER)	53	98%
Progesterone receptor (PR)	45	83%
**Histological grade (Elston and Ellis)**		
Well differentiated (I)	5	9%
Moderately differentiated (II)	31	57%
Poorly differentiated (III)	18	33%

Supplementary Table 2. Immune profile of matched NI and I TDLNs and tumors

Among/CD45	**NI** (Mean + SD)	**I** (Mean + SD)	**T** (Mean + SD)
**B cells**	33,27 + 8,09	38,12 + 6,13	11,56 + 3,51
**Tconv**	51,25 + 8,95	48,2 + 8,07	33,31 + 11,26
**Treg**	0,88 + 0,75	1,38 + 1,32	3,87 + 5,34
**CD8+ T cells**	8,23 + 3,39	10,71 + 6,95	26,16 + 6,76

Supplementary Table 3. TCR beta repertoire of CD4+ T cells from TDLNs and tumors

Tconv

Patient	Sample	Number of sorted CD4+ Tconv cells	TCR beta		
			Total number of reads	Number of clones	**% of shared clones***
1	NI	478993	393624	28246	**1,0**
	I	412819	167364	16214	**10,5**
	T	18294	82181	511	**100,0**
2	NI	796012	606802	40265	**3,4**
	I	613134	162613	18735	**4,5**
	T	17014	82303	1118	**100,0**
3	NI	680475	377915	25178	**0,2**
	I	514570	337532	8805	**0,2**
	T	2637	35796	25	**100,0**
4	NI	333649	2045304	5022	**NA**
	I	245321	73691	6856	**NA**
5	NI	628078	613389	45005	**NA**
	I	279838	142722	21283	**NA**

* among top 100 clones in the tumor

Treg

Patient	Sample	Number of sorted Treg cells	TCR beta		
			Total number of reads	Number of clones	**% of shared clones***
1	NI	15000	226679	1429	**2,6**
	I	25206	357978	3542	**1,9**
	T	2015	63779	73	**100,0**
2	NI	14578	401493	1951	**1,6**
	I	9972	104711	829	**17,5**
	T	500	50831	66	**100,0**
3	NI	10129	70418	492	**NA**
	I	15125	71691	375	**NA**
4	NI	15100	443905	983	**NA**
	I	20059	35986	750	**NA**
5	NI	15062	199055	1966	**NA**
	I	10044	103601	1105	**NA**

* among top 100 clones in the tumor

Supplementary Table 4. TCR beta repertoire overlap of CD4+ T cells from matched TDLNs and tumors

Shared TCRs between Tregs and Tconvs in each tissue							
Tissue	Patients / TCRs	1	2	3	4	5	Mean
NI	# clones	184	449	144	21	408	
	% of Tregs	**12,9**	**23,0**	**29,3**	**2,1**	**20,8**	**17,6**
I	# clones	483	134	54	84	179	
	% of Tregs	**13,6**	**16,2**	**14,4**	**11,2**	**16,2**	**14,3**
T	# clones	14	16	ND	ND	ND	
	% of Tregs	**19,2**	**24,2**	ND	ND	ND	**21,7**

Shared TCR between T-Tregs and TDLN's Tconvs							
Tissue	Parients / TCRs	1	2	3	4	5	Mean
T-Tregs vs NI-Tconvs	# clones	7	16	ND	ND	ND	
	% of Tregs	**9,6**	**24,2**	ND	ND	ND	**16,9**
T-Tregs vs I-Tconvs	# clones	16	18	ND	ND	ND	
	% of Tregs	**16,4**	**27,3**	ND	ND	ND	**21,9**

*The percentage of shared clones was calculated as number of shared clones/ total number of Treg clones x 100, for each tissue.

Supplementary Table 5. Clones and providers of FACs antibodies

Antigens	Clones	Fluorochromes	Dilution	Isotypes	Manufacturers
2B4	C1.7	APC	1/100	mouse IgG1, κ	BD Biosciences
CD27	O323	BV605	1/200	mouse IgG1, κ	BD Biosciences
CD3	OKT3	BV650	1/100	mouse IgG2a, κ	BD Biosciences
CD4	OKT4	BV785	1/150	mouse IgG2b, κ	BD Biosciences
PD1	EH12.2H7	BV711	1/100	mouse IgG1, κ	BD Biosciences
TIM3	F38-2E2	BV421	1/100	mouse IgG1, κ	BD Biosciences
GITR	DT5D3	APC	1/100	mouse IgG1	Miltenyi Biotec
TIM-3	RMT3-23	APC	1/50	mouse IgG1κ	Miltenyi Biotec
KLRG1	REA261	APC-Vio770	1/100	human IgG1	Miltenyi Biotec
CD8	3B5	Alexa 700	1/100	mouse IgG2a, κ	ThermoScientific
EPCAM	1B7	PERCP e710	1/100	mouse IgG1, κ	eBioscience
ICOS	ISA-3	PERCP e710	1/100	mouse IgG1, κ	eBioscience
CD8	2ST8.5H7	ECD	1/50	mouse IgG2a	Beckman Coulter
CD56	N901	PE-Cy5	1/200	mouse IgG1	Beckman Coulter
CD3	UCHT1	PE-Cy7	1/200	mouse IgG1, κ	Beckman Coulter
CD3	UCHT1	Alexa 700	1/100	mouse IgG1, κ	BD Biosciences
CD45	2D1	APC Cy7	1/100	mouse IgG1, κ	BD Biosciences
CD19	HIB19	Alexa 700	1/50	mouse IgG1, κ	BD Biosciences
CD80	L307.4	BV786	1/50	mouse IgG1, κ	BD Biosciences
CD4	M-T466	APC	1/100	mouse IgG1, κ	Miltenyi Biotec
CD45RA	HI100	Alexa 700	1/100	mouse IgG2b, κ	BD Biosciences
CD45RA	HI100	PE-Cy5	1/100	mouse IgG2b, κ	BD Biosciences
OX40	ACT35	PECy5	1/100	mouse IgG1, κ	BD Biosciences
CD25	M-A251	PE	1/20	mouse IgG1, κ	BD Biosciences
CD25	M-A251	APC Cy7	1/20	mouse IgG1, κ	BD Biosciences
CD4	RPA-T4	PE-CF594	1/250	mouse IgG1, κ	BD Biosciences
CCR6	G034E3	BV605	1/150	mouse IgG1, κ	BioLegendTM
CXCR5	51505	APC	1/100	mouse IgG2b, κ	R&D
CXCR3	1C6/CXCR3	PE-CF594	1/50	mouse IgG1, κ	BD Biosciences
CCR4	1G1	PE-Cy7	1/100	mouse IgG1, κ	BD Biosciences
CCR7	GO43H7	BV421	1/80	mouse IgG2a, κ	BioLegendTM
CRTh2	BM16	FITC	1/100	rat IgG2A, k	BioLegendTM
CD127	A019D5	BV650	1/40	mouse IgG1, κ	BioLegendTM
CCR10	FAB3478P	PE	1/100	rat IgG2A	R&D

INTRACELLULAR

Antigens	Clones	Fluorochromes	Dilution	Isotypes	Manufacturers
FOXP3	236A/E7	Alexa 488	1/20	mouse IgG1, κ	eBioscience
FOXP3	236A/E7	PE	1/20	mouse IgG1, κ	eBioscience
FOXP3	259D/C7	PE-CF594	1/20	mouse IgG1, κ	BD Biosciences
GranzymeB	GB11	Alexa647	1/200	mouse IgG1, κ	BD Biosciences
CTLA-4	Goat polyclonal	PE	1/100		R&D
IL17	BL168	BV711	1/100	mouse IgG1, κ	BioLegendTM

IFNγ	B27	V450	1/100	mouse IgG1, κ	BD Biosciences

LIVE/DEAD

Fixable Aqua				ThermoScientific

Supplementary Table 6. TCR beta primers

PCR1 Forward primers

Alpha Forward

TRAV1	CTGCACGTACCAGACATCTGGGTT
TRAV2	GGCTCAAAGCCTTCTCAGCAGG
TRAV3	GGATAACCTGGTTAAAGGCAGCTA
TRAV4	GGATACAAGACAAAAGTTACAAACGA
TRAV5	GCTGACGTATATTTTTTCAAATATGGA
TRAV6	GGAAGAGGCCCTGTTTTCTTGCT
TRAV7	GCTGGATATGAGAAGCAGAAAGGA
TRAV8	AGGACTCCAGCTTCTCCTGAAGTA
TRAV9	GTATGTCCAATATCCTGGAGAAGGT
TRAV10	CAGTGAGAACACAAAGTCGAACGG
TRAV12.1	CCTAAGTTGCTGATGTCCGTATAC
TRAV12.2	GGGAAAAGCCCTGAGTTGATAATGT
TRAV12.3	GCTGATGTACACATACTCCAGTGG
TRAV13.1	CCCTTGGTATAAGCAAGAACTTGG
TRAV13.2	CCTCAATTCATTATAGACATTCGTTC
TRAV14	GCAAAATGCAACAGAAGGTCGCTA
TRAV16	TAGAGAGAGCATCAAAGGCTTCAC
TRAV17	CGTTCAAATGAAAGAGAGAAACACAG
TRAV18	CCTGAAAAGTTCAGAAAACCAGGAG
TRAV19	GGTCGGTATTCTTGGAACTTCCAG
TRAV20	GCTGGGGAAGAAAAGGAGAAAGAAA
TRAV21	GTCAGAGAGAGCAAACAAGTGGAA
TRAV22	GGACAAAACAGAATGGAAGATTAAGC
TRAV23	CCAGATGTGAGTGAAAAGAAAGAAG
TRAV24	GACTTTAAATGGGGATGAAAAGAAGA
TRAV25	GGAGAAGTGAAGAAGCAGAAAAGAC
TRAV26.1	CCAATGAAATGGCCTCTCTGATCA
TRAV26.2	GCAATGTGAACAACAGAATGGCCT
TRAV27	GGTGGAGAAGTGAAGAAGCTGAAG
TRAV29	GGATAAAAATGAAGATGGAAGATTCAC
TRAV30	CCTGATGATATTACTGAAGGGTGGA
TRAV34	GGTGGGGAAGAGAAAAGTCATGAA
TRAV35	GGTGAATTGACCTCAAATGGAAGAC
TRAV36	GCTAACTTCAAGTGGAATTGAAAAGA
TRAV38	GAAGCTTATAAGCAACAGAATGCAAC
TRAV39	GGAGCAGTGAAGCAGGAGGGAC
TRAV40	GAGAGACAATGGAAAACAGCAAAAAC
TRAV41	GCTGAGCTCAGGGAAGAAGAAGC

Beta Forward

TRBV2	CTGAAATATTCGATGATCAATTCTCAG
TRBV3-1	TCATTATAAATGAAACAGTTCCAAATCG
TRBV4	AGTGTGCCAAGTCGCTTCTCAC
TRBV5-4,8	CAGAGGAAACTYCCCTCCTAGATT
TRBV5-1	GAGACACAGAGAAACAAAGGAAACTTC
TRBV6-1	GGTACCACTGACAAAGGAGAAGTCC
TRBV6-2,3	GAGGGTACAACTGCCAAAGGAGAGGT
TRBV6-4	GGCAAAGGAGAAGTCCCTGATGGTT
TRBV6-5,6	AAGGAGAAGTCCCSAATGGCTACAA
TRBV6-8	CTGACAAAGAAGTCCCCAATGGCTAC
TRBV6-9	CACTGACAAAGGAGAAGTCCCCGAT
TRBV7-2	AGACAAATCAGGGCTGCCCAGTGA
TRBV7-3	GACTCAGGGCTGCCCAACGAT
TRBV7-8	CCAGAATGAAGCTCAACTAGACAA
TRBV7-4,6	GGTTCTCTGCAGAGAGGCCTGAG
TRBV7-7	GGCTGCCCAGTGATCGGTTCTC
TRBV7-9	GACTTACTTCCAGAATGAAGCTCAACT
TRBV9	GAGCAAAAGGAAACATTCTTGAACGATT
TRBV10-1,3	GGCTRATCCATTACTCATATGGTGTT
TRBV10-2	GATAAAGGAGAAGTCCCCGATGGCT
TRBV11	GATTCACAGTTGCCTAAGGATCGAT
TRBV12-3,4	GATTCAGGGATGCCCGAGGATCG
TRBV12-5	GATTCGGGGATGCCGAAGGATCG
TRBV13	GCAGAGCGATAAAGGAAGCATCCCT
TRBV14	TCCGGTATGCCCAACAATCGATTCT
TRBV15	GATTTTAACAATGAAGCAGACACCCCT
TRBV16	GATGAAACAGGTATGCCCAAGGAAAG
TRBV18	TATCATAGATGAGTCAGGAATGCCAAAG
TRBV19	GACTTTCAGAAAGGAGATATAGCTGAA
TRBV20-1	CAAGGCCACATACGAGCAAGGCGTC
TRBV24-1	CAAAGATATAAACAAAGGAGAGATCTCT
TRBV25-1	AGAGAAGGGAGATCTTTCCTCTGAGT
TRBV27-1	GACTGATAAGGGAGATGTTCCTGAAG
TRBV28	GGCTGATCTATTTCTCATATGATGTTAA
TRBV29	GCCACATATGAGAGTGGATTTGTCATT
TRBV30	GGTGCCCCAGAATCTCTCAGCCT

PCR2 Forward primers

Nested alpha forward 2 with adaptor CS1 (in red)

TRAV1	ACACTGACGACATGGTTCTACAAGGTCGTTTTTCTTCATTCCTTAGTC
TRAV2	ACACTGACGACATGGTTCTACAACGATACAACATGACCTATGAACGG
TRAV3.1	ACACTGACGACATGGTTCTACACTTTGAAGCTGAATTTAACAAGAGCC
TRAV4.1	ACACTGACGACATGGTTCTACACTCCCTGTTTATCCCTGCCGAC
TRAV5.1	ACACTGACGACATGGTTCTACAAAACAAGACCAAAGACTCACTGTTC
TRAV6	ACACTGACGACATGGTTCTACAAAGACTGAAGGTCACCTTTGATACC
TRAV7	ACACTGACGACATGGTTCTACAACTAAATGCTACATTACTGAAGAATGG
TRAV8	ACACTGACGACATGGTTCTACAGCATCAACGGTTTTGAGGCTGAATTTAA
TRAV9	ACACTGACGACATGGTTCTACAGAAACCACTTCTTTCCACTTGGAGAA
TRAV10	ACACTGACGACATGGTTCTACATACAGCAACTCTGGATGCAGACAC
TRAV12	ACACTGACGACATGGTTCTACAGAAGATGGAAGGTTTACAGCACA
TRAV13.1	ACACTGACGACATGGTTCTACAGACATTCGTTCAAATGTGGGCGAA
TRAV13.2	ACACTGACGACATGGTTCTACAGGCAAGGCCAAAGAGTCACCGT
TRAV14	ACACTGACGACATGGTTCTACATCCAGAAGGCAAGAAAATCCGCCA
TRAV16	ACACTGACGACATGGTTCTACAGCTGACCTTAACAAAGGCGAGACA
TRAV17	ACACTGACGACATGGTTCTACATTAAGAGTCACGCTTGACACTTCCA
TRAV18	ACACTGACGACATGGTTCTACAGCAGAGGTTTTCAGGCCAGTCCT
TRAV19	ACACTGACGACATGGTTCTACATCCACCAGTTCCTTCAACTTCACC
TRAV20	ACACTGACGACATGGTTCTACAGCCACATTAACAAAGAAGGAAAGCT
TRAV21	ACACTGACGACATGGTTCTACAGCCTCGCTGGATAAATCATCAGGA
TRAV22	ACACTGACGACATGGTTCTACAACGACTGTCGCTACGGAACGCTA
TRAV23	ACACTGACGACATGGTTCTACACACAATCTCCTTCAATAAAAGTGCCA
TRAV24	ACACTGACGACATGGTTCTACAACGAATAAGTGCCACTCTTAATACCA
TRAV25	ACACTGACGACATGGTTCTACAGTTTGGAGAAGCAAAAAAGAACAGCT
TRAV26.1	ACACTGACGACATGGTTCTACACAGAAGACAGAAAGTCCAGCACCT
TRAV26.2	ACACTGACGACATGGTTCTACAATCGCTGAAGACAGAAAGTCCAGT
TRAV27	ACACTGACGACATGGTTCTACAACTAACCTTTCAGTTTGGTGATGCAA
TRAV29	ACACTGACGACATGGTTCTACACTTAAACAAAAGTGCCAAGCACCTC
TRAV30	ACACTGACGACATGGTTCTACAAATATCTGCTTCATTTAATGAAAAAAAGC
TRAV34	ACACTGACGACATGGTTCTACACCAAGTTGGATGAGAAAAAGCAGCA
TRAV35	ACACTGACGACATGGTTCTACACTCAGTTTGGTATAACCAGAAAGGA
TRAV36	ACACTGACGACATGGTTCTACAGGAAGACTAAGTAGCATATTAGATAAG
TRAV38	ACACTGACGACATGGTTCTACACTGTGAACTTCCAGAAAGCAGCCA
TRAV39	ACACTGACGACATGGTTCTACACCTCACTTGATACCAAAGCCCGT
TRAV40	ACACTGACGACATGGTTCTACAAGGCGGAAATATTAAAGACAAAAACTC
TRAV41	ACACTGACGACATGGTTCTACAGATTAATTGCCACAATAAACATACAGG

Nested beta forward 2 with adaptor CS1 (in red)

TRBV2	ACACTGACGACATGGTTCTACAGCCTGATGGATCAAATTTCACTCTG
TRBV3-1	ACACTGACGACATGGTTCTACATCTCACCTAAATCTCCAGACAAAGCT
TRBV4	ACACTGACGACATGGTTCTACACCTGAATGCCCCAACAGCTCTC
TRBV5-4,8	ACACTGACGACATGGTTCTACACTCTGAGCTGAATGTGAACGCCT
TRBV5-1	ACACTGACGACATGGTTCTACACGATTCTCAGGGCGCCAGTTCTCT
TRBV6-1	ACACTGACGACATGGTTCTACATGGCTACAATGTCTCCAGATTAAACAA
TRBV6-2,3	ACACTGACGACATGGTTCTACACCCTGATGGCTACAATGTCTCCAGA
TRBV6-4	ACACTGACGACATGGTTCTACAGTGTCTCCAGAGCAAACACAGATGATT
TRBV6-5,6	ACACTGACGACATGGTTCTACAGTCTCCAGATCAACCACAGAGGAT
TRBV6-8	ACACTGACGACATGGTTCTACAGTCTCTAGATTAAACACAGAGGATTTC
TRBV6-9	ACACTGACGACATGGTTCTACAGGCTACAATGTATCCAGATCAAACA
TRBV7-2	ACACTGACGACATGGTTCTACATCGCTTCTCTGCAGAGAGGACTGG
TRBV7-3	ACACTGACGACATGGTTCTACACGGTTCTTTGCAGTCAGGCCTGA
TRBV7-8	ACACTGACGACATGGTTCTACACCAGTGATCGCTTCTTTGCAGAAA
TRBV7-4,6	ACACTGACGACATGGTTCTACATCTCCACTCTGAMGATCCAGCGCA
TRBV7-7	ACACTGACGACATGGTTCTACAGCAGAGAGGCCTGAGGGATCCAT
TRBV7-9	ACACTGACGACATGGTTCTACACTGCAGAGAGGCCTAAGGGATCT
TRBV9	ACACTGACGACATGGTTCTACACTCCGCACAACAGTTCCCTGACTT
TRBV10-1,3	ACACTGACGACATGGTTCTACACAGATGGCTAYAGTGTCTCTAGATCAAA
TRBV10-2	ACACTGACGACATGGTTCTACAGTTGTCTCCAGATCCAAGACAGAGAA
TRBV11	ACACTGACGACATGGTTCTACAGCAGAGAGGCTCAAAGGAGTAGACT
TRBV12-3,4	ACACTGACGACATGGTTCTACAGCTAAGATGCCTAATGCATCATTCTC
TRBV12-5	ACACTGACGACATGGTTCTACACTCAGCAGAGATGCCTGATGCAACT
TRBV13	ACACTGACGACATGGTTCTACATCTCAGCTCAACAGTTCAGTGACTA
TRBV14	ACACTGACGACATGGTTCTACAGCTGAAAGGACTGGAGGGACGTAT
TRBV15	ACACTGACGACATGGTTCTACAGATAACTTCCAATCCAGGAGGCCG
TRBV16	ACACTGACGACATGGTTCTACAGCTAAGTGCCTCCCAAATTCACCC
TRBV18	ACACTGACGACATGGTTCTACAGGAACGATTTTCTGCTGAATTTCCCA
TRBV19	ACACTGACGACATGGTTCTACAGGTACAGCGTCTCTCGGGAGAAGA
TRBV20-1	ACACTGACGACATGGTTCTACAGGACAAGTTTCTCATCAACCATGCAA
TRBV24-1	ACACTGACGACATGGTTCTACATGGATACAGTGTCTCTCGACAGGC
TRBV25-1	ACACTGACGACATGGTTCTACACAACAGTCTCCAGAATAAGGACGGA
TRBV27-1	ACACTGACGACATGGTTCTACATACAAAGTCTCTCGAAAAGAGAAGAGGA
TRBV28	ACACTGACGACATGGTTCTACAGGGGTACAGTGTCTCTAGAGAGA
TRBV29	ACACTGACGACATGGTTCTACAGTTTCCCATCAGCCGCCCAAACCTA
TRBV30	ACACTGACGACATGGTTCTACACAGACCCCAGGACCGGCAGTTCAT

PCR1 & 2 Reverse primers

Alpha Reverse with CS2 adaptor (in orange)

CS2-TRAC	TACGGTAGCAGAGACTTGGTCTTCGGTGAATAGGCAGACAGACTTGT

Beta Reverse with CS2 adaptor (in orange)

CS2-TRBC	TACGGTAGCAGAGACTTGGTCTTACCAGTGTGGCCTTTTGGGTGTG
