# Supplementary material for: Tumor invasion in draining lymph nodes is associated with Treg accumulation in breast cancer patients
Source: Nat Commun. 2020 Jun 29;11:3272. doi: 10.1038/s41467-020-17046-2 (PMC7324591; doi:10.1038/s41467-020-17046-2)
Supplement: Supplementary file 3 — Descriptions of Additional Supplementary Files [file 41467_2020_17046_MOESM3_ESM.pdf]

Descriptions of Additional Supplementary Files

File name: Supplementary Data 1

Description: Differential gene expression analysis of T cells from NI and I TDLNs, and tumor. Differentially expressed genes table (DEGs, fold change 1.2, $p<0.05$) between Tregs and Tconvs in NI (2985) TDLNs, I (868) TDLNs and tumors (1800).

File name: Supplementary Data 2

Description: Canonical Pathway Analysis of T cells from NI and I TDLNs, and tumor. Canonical Ingenuity Pathway (IPA) table of differentially expressed.

File name: Supplementary Data 3

Description: EnrichR analysis of differentially expressed genes. Differentially expressed genes (DEGs, fold change 1.2, $p<0.05$) between Tregs and Tconvs in NI (2985) TDLNs, I (868) TDLNs and tumors (1800).
