# Supplementary material for: Tumor invasion in draining lymph nodes is associated with Treg accumulation in breast cancer patients
Source: Nat Commun. 2020 Jun 29;11:3272. doi: 10.1038/s41467-020-17046-2 (PMC7324591; doi:10.1038/s41467-020-17046-2)
Supplement: Supplementary file 7 — Reporting Summary [file 41467_2020_17046_MOESM7_ESM.pdf]

nature research

Corresponding author(s): Eliane Piaggio

Last updated by author(s): May 28, 2020

Reporting Summary

Nature Research wishes to improve the reproducibility of the work that we publish. This form provides structure for consistency and transparency in reporting. For further information on Nature Research policies, see Authors & Referees and the Editorial Policy Checklist .

Statistics

For all statistical analyses, confirm that the following items are present in the figure legend, table legend, main text, or Methods section.

n/a	Confirmed	
☐	☒	The exact sample size (*n*) for each experimental group/condition, given as a discrete number and unit of measurement
☐	☒	A statement on whether measurements were taken from distinct samples or whether the same sample was measured repeatedly
☐	☒	The statistical test(s) used AND whether they are one- or two-sided<br>*Only common tests should be described solely by name; describe more complex techniques in the Methods section.*
☒	☐	A description of all covariates tested
☒	☐	A description of any assumptions or corrections, such as tests of normality and adjustment for multiple comparisons
☐	☒	A full description of the statistical parameters including central tendency (e.g. means) or other basic estimates (e.g. regression coefficient) AND variation (e.g. standard deviation) or associated estimates of uncertainty (e.g. confidence intervals)
☐	☒	For null hypothesis testing, the test statistic (e.g. *F*, *t*, *r*) with confidence intervals, effect sizes, degrees of freedom and *P* value noted<br>*Give P values as exact values whenever suitable.*
☒	☐	For Bayesian analysis, information on the choice of priors and Markov chain Monte Carlo settings
☒	☐	For hierarchical and complex designs, identification of the appropriate level for tests and full reporting of outcomes
☒	☐	Estimates of effect sizes (e.g. Cohen's *d*, Pearson's *r*), indicating how they were calculated

Our web collection on statistics for biologists contains articles on many of the points above.

Software and code

Policy information about availability of computer code

Data collection	BD FACSDiva software - Fortessa flow cytometer (BD Biosciences) - BD FACS ARIA II cell sorter (BD Biosciences)
Data analysis	FlowJo V10, Prism 8.0 software (GraphPad), R studio Version 1.2.1335 - TCGA-BRCA<br>EnrichR

For manuscripts utilizing custom algorithms or software that are central to the research but not yet described in published literature, software must be made available to editors/reviewers. We strongly encourage code deposition in a community repository (e.g. GitHub). See the Nature Research guidelines for submitting code & software for further information.

Data

Policy information about availability of data

All manuscripts must include a data availability statement. This statement should provide the following information, where applicable:

- Accession codes, unique identifiers, or web links for publicly available datasets
- A list of figures that have associated raw data
- A description of any restrictions on data availability

The RNA-seq data are available at https://www.ebi.ac.uk/arrayexpress/experiments/E-MTAB-9112 - TCR-seq data that support the findings of this study have been deposited in NCBI Gene Expression Omnibus with the GSE115545 accession code: [https://www.ncbi.nlm.nih.gov/geo/query/acc.cgi?acc=GSE115545] - TCGA-BRCA (Breast Cancer) - Legacy -https://portal.gdc.cancer.gov/legacy-archive - mRNA gene expression and quantification HT_HG-U133A

Field-specific reporting

Please select the one below that is the best fit for your research. If you are not sure, read the appropriate sections before making your selection.

Life sciences study design

All studies must disclose on these points even when the disclosure is negative.

Sample size	No sample size calculation was performed. We used all the samples available in each dataset.
Data exclusions	No exclusions. All experimental results were included.
Replication	All experiments were performed at least twice and reproducible results were obtained.
Randomization	No randomisation was used. Since we mainly perform pair-wise comparisons, variables like age, and genetic differences do not confound our analyses. In order to avoid batch effects, the matched organs were always processed in the same batch.
Blinding	The investigators handling the samples were not blinded during data collection and processing as the nature of the organ (TDLNs and tumor) was visibly different.

Reporting for specific materials, systems and methods

We require information from authors about some types of materials, experimental systems and methods used in many studies. Here, indicate whether each material, system or method listed is relevant to your study. If you are not sure if a list item applies to your research, read the appropriate section before selecting a response.

Materials & experimental systems

n/a	Involved in the study	
☐	☒	Antibodies
☒	☐	Eukaryotic cell lines
☒	☐	Palaeontology
☒	☐	Animals and other organisms
☐	☒	Human research participants
☒	☐	Clinical data

Methods

n/a	Involved in the study	
☒	☐	ChIP-seq
☐	☒	Flow cytometry
☒	☐	MRI-based neuroimaging

Antibodies

Antibodies used

Antigens Clones Fluorochromes Isotypes Manufacturers
CD32 IV.3 purified mouse IgG2b, κ Stem Cell
2B4 C1.7 APC mouse IgG1, κ BD Biosciences
CD27 O323 BV605 mouse IgG1, κ BD Biosciences
CD3 OKT3 BV650 mouse IgG2a, κ BD Biosciences
CD4 OKT4 BV785 mouse IgG2b, κ BD Biosciences
PD1 EH12.2H7 BV711 mouse IgG1, κ BD Biosciences
TIM3 F38-2E2 BV421 mouse IgG1, κ BD Biosciences
GITR DT5D3 APC mouse IgG1 Miltenyi Biotec
CD4 M-T466 APC mouse IgG1 Miltenyi Biotec
TIM-3 RMT3-23 APC mouse IgG1κ Miltenyi Biotec
KLRG1 REA261 APC-Vio770 human IgG1 Miltenyi Biotec
CD8 3B5 Alexa 700 mouse IgG2a, κ ThermoScientific
EPCAM 1B7 PERCP e710 mouse IgG1, κ eBioscience
ICOS ISA-3 PERCP e710 mouse IgG1, κ eBioscience
CD8 2ST8.5H7 ECD mouse IgG2a Beckman Coulter
CD56 N901 PE-Cy5 mouse IgG1 Beckman Coulter
CD3 UCHT1 PE-Cy7 mouse IgG1, κ Beckman Coulter
CD3 UCHT1 Alexa 700 mouse IgG1, κ BD Biosciences
CD45 2D1 APC Cy7 mouse IgG1, κ BD Biosciences
CD19 HIB19 Alexa 700 mouse IgG1, κ BD Biosciences
CD45RA HI100 Alexa 700 mouse IgG2b, κ BD Biosciences
CD45RA HI100 PE-Cy5 mouse IgG2b, κ BD Biosciences
OX40 ACT35 PECy5 mouse IgG1, κ BD Biosciences
CD25 M-A251 PE mouse IgG1, κ BD Biosciences
CD25 M-A251 APC Cy7 mouse IgG1, κ BD Biosciences
CD4 RPA-T4 PE-CF594 mouse IgG1, κ BD Biosciences
CCR6 G034E3 BV605 mouse IgG1, κ BioLegendTM
CXCR5 51505 APC mouse IgG2b, κ R&D

CXCR3 1C6/CXCR3 PE-CF594 mouse IgG1, κ BD Biosciences
CCR4 1G1 PE-Cy7 mouse IgG1, κ BD Biosciences
CCR7 GO43H7 BV421 mouse IgG2a, κ BioLegendTM
CRTh2 BM16 FITC rat IgG2A, k BioLegendTM
CD127 A019D5 BV650 mouse IgG1, κ BioLegendTM
CCR10 FAB3478P PE rat IgG2A R&D

INTRACELLULAR
Antigens Clones Fluorochromes Manufacturers
FOXP3 236A/E7 Alexa 488 mouse IgG1, κ eBioscience
FOXP3 236A/E7 PE mouse IgG1, κ eBioscience
FOXP3 259D/C7 PE-CF594 mouse IgG1, κ BD Biosciences
GranzymeB GB11 Alexa647 mouse IgG1, κ BD Biosciences
CTLA-4 Goat polyclonal PE R&D
IL17 BL168 BV711 mouse IgG1, κ BioLegendTM
IFNγ B27 V450 mouse IgG1, κ BD Biosciences
CD80 L307.4 BV786 mouse IgG1k BD Biosciences
IgG1k clone X40 BV786 BD Biosciences

LIVE/DEAD Fixable Aqua ThermoScientific

Validation: All the antibodies have been validated by the manufacturer and then titrated in house.

Human research participants

Policy information about studies involving human research participants

Population characteristics: TDLNs and tumors were collected from 54 patients with an average of 57 years with luminal breast cancer having undergone standard-of-care surgical resection. The clinical and pathological characteristic are described in Supplementary Table 1.

Recruitment: The study cohort included volunteer patients for whom samples of the NI and I TDLNs and the primary tumor were available at the institut Curie Hospital (Paris, France).

Ethics oversight: TDLNs and tumors were collected at the Institut Curie Hospital (Paris, France), in accordance with institutional ethical guidelines and informed consent was obtained. The protocol was approved by the Ethical Committee of Curie Institut ("Comité de la Recherche Institutionnel", CRI-0804-2015).

Note that full information on the approval of the study protocol must also be provided in the manuscript.

Flow Cytometry

Plots

Confirm that:

- [x] The axis labels state the marker and fluorochrome used (e.g. CD4-FITC).
- [x] The axis scales are clearly visible. Include numbers along axes only for bottom left plot of group (a 'group' is an analysis of identical markers).
- [x] All plots are contour plots with outliers or pseudocolor plots.
- [x] A numerical value for number of cells or percentage (with statistics) is provided.

Methodology

Sample preparation: Samples were obtained within a few hours after the primary surgery, cut into small fragments, and digested with 0.1 mg/ml Liberase TL (Roche) in the presence of 0.1 mg/ml DNase (Roche) for 20 min before the addition of 10 mM EDTA. Cells were filtered on a 40-µm cell strainer (BD Biosciences).

Instrument: BD FACSDiva software - Fortessa flow cytometer (BD Biosciences) - BD FACS ARIA II cell sorter (BD Biosciences)

Software: FlowJo V10

Cell population abundance: Expressed as a frequency of the selected population

Gating strategy: The FMOs were used for determining the division point for negative and positive population

- [x] Tick this box to confirm that a figure exemplifying the gating strategy is provided in the Supplementary Information.
